# Case Report: Acute myeloid leukemia with mixed immunophenotypic features and TCF3::ZNF384 fusion in a pediatric patient with CNS involvement: a diagnostic and therapeutic challenge

**DOI:** 10.3389/fped.2026.1766046

**Published:** 2026-05-28

**Authors:** Aser Alonso-Carballo, Natalia Mateos, Paula Gómez, Sara Montolio, Jose Antonio Salinas, Antonio Gutiérrez, Antonia Sampol, Jordi Martínez-Serra

**Affiliations:** 1 Hematology Department, Son Espases University Hospital, IdISBa, Palma, Spain; 2 Pediatrics Department, Son Espases University Hospital, IdISBa, Palma, Spain

**Keywords:** lineage plasticity, minimal residual disease, mixed-phenotype acute leukemia, next-generation sequencing, pediatric acute leukemia, TCF3::ZNF384 fusion

## Abstract

Acute myeloid leukemia (AML) in the pediatric population can present significant diagnostic and therapeutic challenges, especially when immunophenotypic or genetic findings indicate mixed lineage features. Here, we present the case of an 11-year-old female patient who was initially diagnosed with AML (FLT3-ITD negative) and central nervous system (CNS-3) involvement. The leukemic blasts co-expressed myeloid markers (MPO, CD33, and CD13) as well as a low-intensity B-lymphoid antigen (CD19). Despite receiving two standard induction regimens (MEC and ADE), minimal residual disease (MRD) persisted. Next-generation sequencing (Illumina MiSeq; SOPHiA DDM Myeloid Plus) was performed on bone marrow aspirate collected after the second induction cycle (ADE), at which point MRD had become negative by flow cytometry but persistent MRD had been documented after the first induction (MEC). Post-induction NGS revealed a TCF3::ZNF384 fusion with a fusion read support of 75.04% (supported by 21,262 reads and 80.56% unique molecular coverage). This aberration is more commonly associated with B-ALL or mixed-phenotype acute leukemia (MPAL-B/M), which are both characterized by lineage plasticity and a higher risk of relapse. These results highlight the importance of comprehensive molecular testing and the need for therapeutic strategies that integrate both myeloid- and lymphoid-directed approaches, as well as meticulous MRD surveillance.

## 1 Introduction

Acute myeloid leukemia (AML) accounts for 15%–20% of childhood acute leukemias. It is associated with poorer outcomes than acute lymphoblastic leukemia (ALL), primarily due to relapse and treatment resistance (1). Recent advances in flow cytometry and next-generation sequencing (NGS) have significantly improved characterization of leukemias with ambiguous or mixed immunophenotypic features, refining diagnosis and guiding risk-adapted therapy (2).

Genetic alterations that are increasingly recognized in mixed phenotype acute leukemia (MPAL) and atypical AML presentations include ZNF384 rearrangements, particularly the TCF3::ZNF384 fusion (3). This lesion occurs in approximately 1%–2% of childhood ALL cases and up to 40% of MPAL cases with a B/myeloid phenotype. Leukemias harboring ZNF384 fusions typically exhibit a characteristic immunophenotype: low or negative CD10 expression; frequent aberrant expression of myeloid markers (CD13, CD33); and occasional myeloperoxidase (MPO) positivity. This can lead to misclassification as AML at diagnosis (4, 5). According to the WHO 2022 and ICC 2022 classifications, MPAL is defined by the co-expression of markers from two distinct lineages (myeloid and lymphoid) on the same blast population, or by two distinct blast populations each expressing lineage-specific markers. The molecular pathogenesis of MPAL is heterogeneous: ZNF384-rearranged cases, including TCF3::ZNF384, represent a biologically distinct subtype characterized by aberrant activation of hematopoietic transcription factors, epigenetic dysregulation, and the acquisition of lineage-ambiguous differentiation programs. These molecular features underpin the immunophenotypic plasticity and the diagnostic challenges inherent to this entity (4).

The TCF3::ZNF384 fusion gene arises from a translocation involving TCF3 (19p13.3) and ZNF384 (12p13.31) (6). Functional studies have demonstrated that this chimeric oncoprotein disrupts hematopoietic transcriptional programs and promotes lineage plasticity, thereby facilitating bidirectional lineage conversion between the myeloid and lymphoid compartments (7). Clinically, this results in dynamic immunophenotypic shifts and a tendency for lineage switching during therapy or relapse.

Identifying TCF3::ZNF384 and other ZNF384 fusions has significant therapeutic implications (8). While most patients present with B-lineage features and receive ALL-directed treatment, a subset, particularly those misclassified as AML, may require combined or sequential AML/ALL regimens to achieve durable remission. Furthermore, these leukemias often exhibit activation of pathways, such as FLT3 signaling, which could represent a target for intervention (9).

Given their biological heterogeneity, ZNF384-rearranged leukemias challenge the conventional binary classification of AML vs. ALL (10). Integrating immunophenotypic, cytogenetic, and molecular data is therefore essential to accurately diagnose and manage these cases, especially in children presenting with atypical features or CNS involvement. The present case study illustrates the diagnostic and therapeutic complexities encountered in an 11-year-old patient who was initially diagnosed with AML but was later found to have a TCF3::ZNF384 fusion. This case highlights the clinical importance of early RNA-based fusion testing in atypical AML presenting with a minor B-lineage population and CNS disease.

## 2 Case presentation

### 2.1 Clinical presentation

An 11-year-old female patient presented with neurological symptoms, including mild facial nerve palsy. Brain MRI revealed meningeal thickening consistent with CNS infiltration (CNS-3 status, defined as ≥5 WBC/*μ*L with blasts in the cerebrospinal fluid). Peripheral blood smears revealed approximately 70% blasts that expressed myeloid markers (MPO, CD33, CD13) as well as low-intensity CD19 expression. A subset of blasts displayed monocytic features (CD64, CD15), suggesting AML with aberrant B-lineage antigen expression.

### 2.2 Immunophenotypic characterization at diagnosis

**Figure 1:**
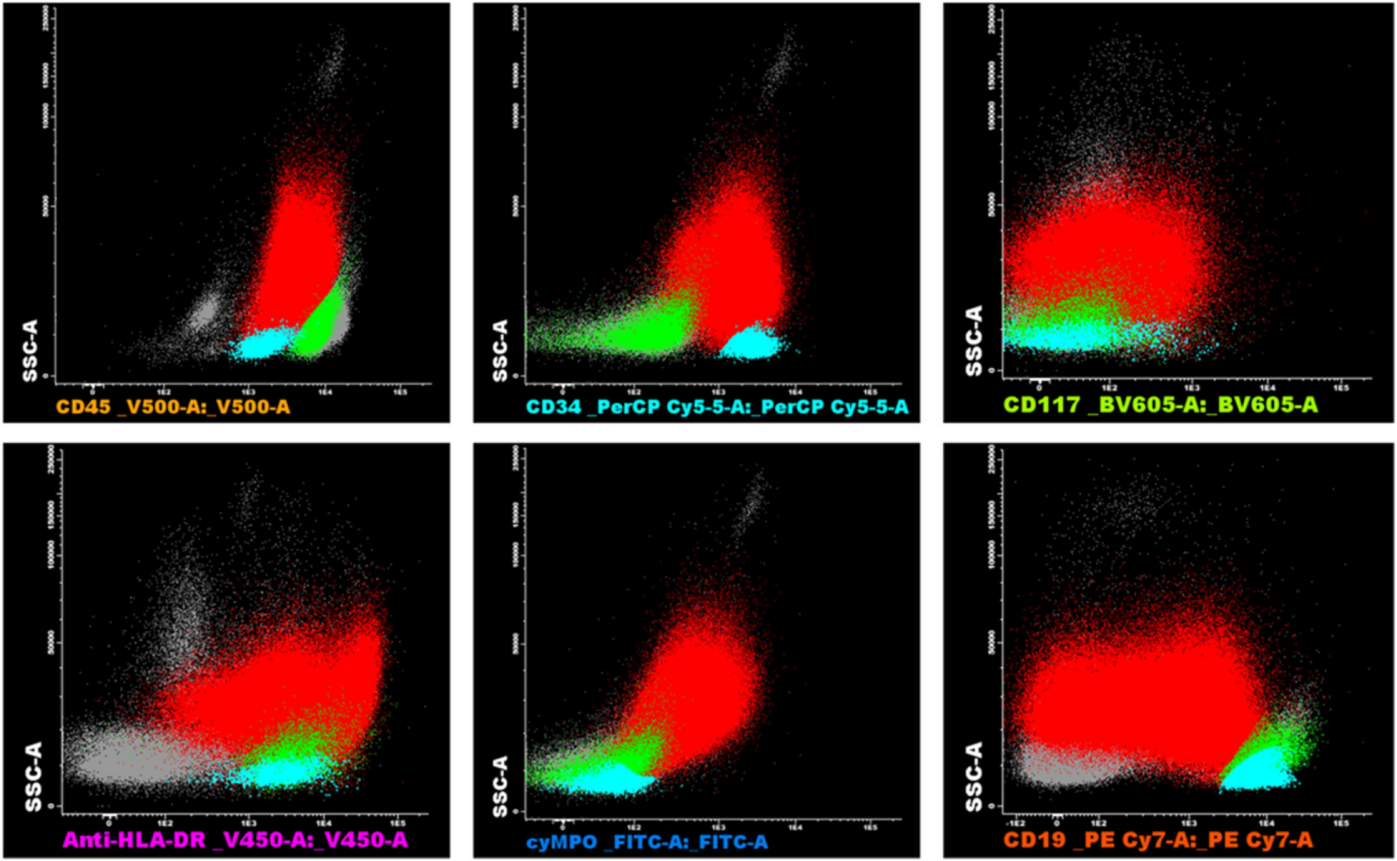
Lineage and maturation markers: CD45, CD34, CD117, HLA-DR, cyMPO, and CD19. The dominant blast population (red) defines the CD45+/++ gate with low SSC-A. CD34++ confirms hematopoietic immaturity. CD117 is absent. HLA-DR is strongly overexpressed (HLA-DR++/+++). cyMPO positivity establishes myeloid lineage commitment. CD19 is aberrantly co-expressed on myeloid blasts; the minor population (cyan) displays CD19++.

**Figure 2:**
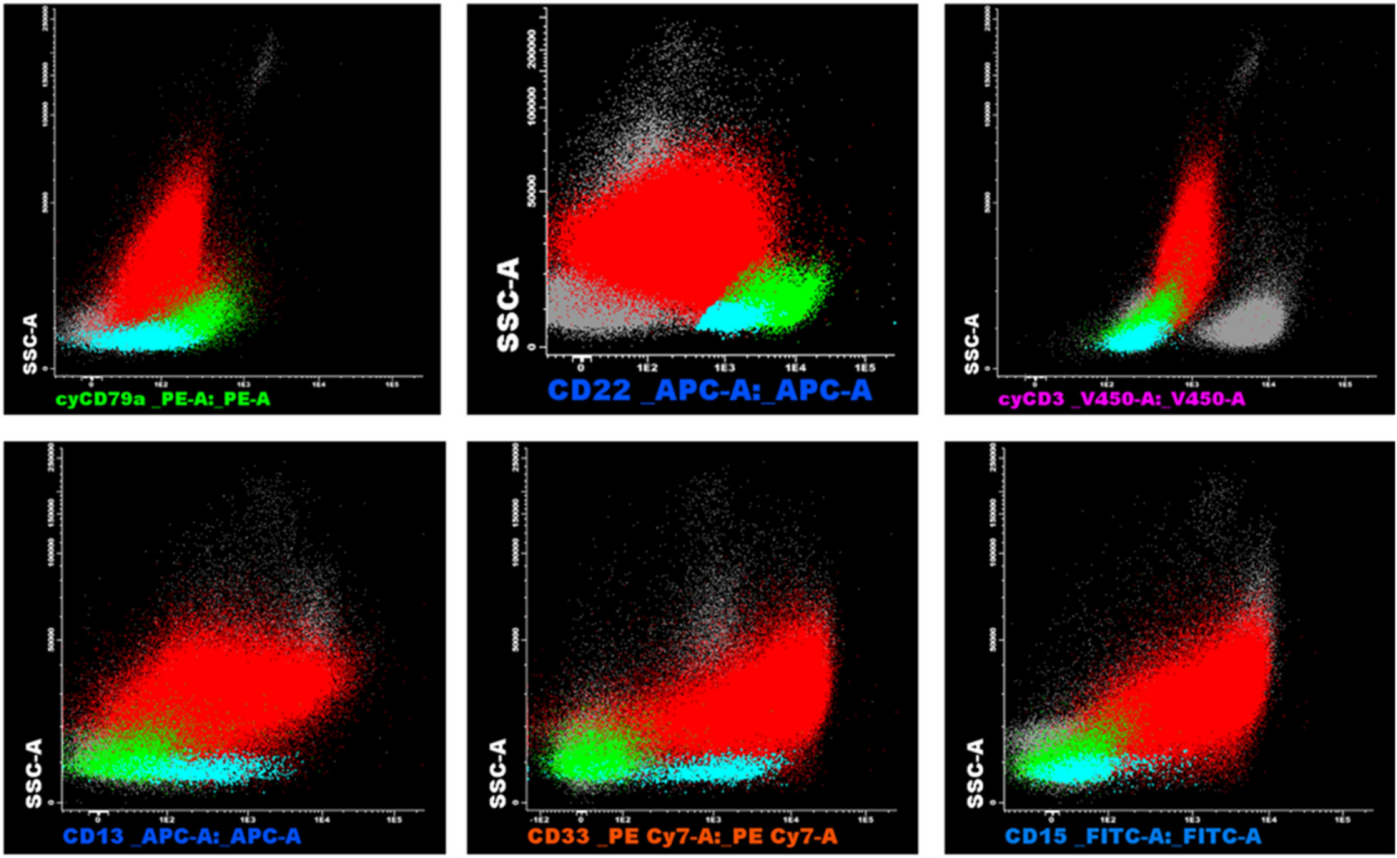
B-lineage and myeloid differentiation markers: cyCD79a, CD22, cyCD3, CD13, CD33, and CD15. CD33++ strongly confirms myeloid lineage on the dominant blast population (red). cyCD79a negativity and partial CD22 expression (CD22−/+) exclude true B-lymphoid lineage commitment, establishing the aberrant co-expression phenotype. cyCD3 negativity rules out T-lineage involvement. CD15 marks the monocytic subset (green).

Multiparameter flow cytometry was performed on bone marrow aspirate using a comprehensive panel of monoclonal antibodies conjugated to different fluorochromes. Dot plots of side scatter (SSC-A) vs. fluorescence intensity are shown in Figures 1, 2. Four color-coded populations are identified throughout: dominant blast population (red), monocytic subset (green), erythroid precursors and minor blast population (cyan), and residual normal cells (gray).

### 2.3 Initial molecular characterization

Initial molecular testing was negative for recurrent AML- and ALL-associated mutations or rearrangements, including FLT3-ITD, KMT2A, NPM1, PML-RARA, and BCR-ABL1. Conventional karyotyping was inconclusive due to an insufficient number of metaphases, while targeted fluorescence *in situ* hybridization (FISH) ruled out rearrangements involving KMT2A, RUNX1, and inv(16). Notably, a partial deletion at the CBFB locus was detected in most analyzed cells, though there was no evidence of the canonical CBFB::MYH11 fusion. The clinical significance of this finding was uncertain, as it was considered a potential technical artifact or secondary aberration.

### 2.4 Treatment course and clinical evolution

#### 2.4.1 Induction

The patient underwent the first induction with MEC protocol (mitoxantrone, etoposide and cytarabine) along with intrathecal prophylaxis, with a good response observed in the bone marrow evaluation on day +22, and flow cytometry MRD (with a sensitivity of 10⁻⁴) was 4.9%. The patient underwent two additional bone marrow aspirate evaluations prior to the second induction block (D + 29 and D + 36), highlighting the negativization of the myeloid clone and the persistence of positive MRD (0.83%) for the minor clone, now expressing CD22++ (weak expression at diagnosis) and CD19++ and therefore now meeting B line criteria.

Following the second induction cycle with ADE (cytarabine, daunorubicin and etoposide) and intrathecal prophylaxis, the bone marrow re-evaluation had a negative MRD result. Repeated cerebrospinal fluid analyses were negative for blasts, and CNS findings were clinically stable.

#### 2.4.2 Infectious complications

The patient developed several infectious complications during marrow aplasia following chemotherapy, including probable fungal pneumonia, suspected endocarditis, and transient viral and bacterial infections. These were all successfully managed with broad-spectrum antimicrobial and antifungal therapy.

#### 2.4.3 Diagnostic reconsideration

Given that the diagnostic presentation was not entirely compatible with acute myeloid leukemia and the patient never presented >5% blasts that met B lineage criteria after induction with MEC and ADE protocols, it was decided to continue with the myeloid treatment regimen (NOPHO-AML-2012 protocol; Nordic Society of Paediatric Haematology and Oncology AML 2012 protocol).

#### 2.4.4 Consolidation and current Status

Subsequently, the patient received an initial consolidation chemotherapy block with the HAM (high-dose cytarabine and mitoxantrone) regimen, completed with two additional consolidation blocks using the HA3E (high-dose cytarabine and etoposide) and FLA (fludarabine and cytarabine) regimens, maintaining complete remission.

The patient has been maintained with negative MRD since the end of induction and is currently in the 5th month of follow-up post-treatment completion.

A comprehensive overview of key clinical, laboratory, and treatment milestones is provided in Table 1.

**Table 1:** Chronological overview of clinical events, laboratory findings, treatment course, and MRD monitoring.

Phase	Day	CBC/PB Blasts	BM Blasts (%)	CSF (WBC/μL + blasts)	Clinical Event	MRD (%)	Notes/Molecular
DIAGNOSIS							
Diagnosis	D0	WBC: 35,800/μL Hb: 5.2 g/dL Plt: 70,000/μL PB blasts: 70%	-	CNS-3 < 2 WBC/μL No blasts	Facial nerve palsy Brain MRI: meningeal thickening Stroke due to middle cerebral artery occlusion, left hemiparesis MEC D1 + IT prophylaxis started	-	FC: (84%) MPO+, CD33++, CD19±; (0.5%) CD19++, CD22± FLT3-ITD neg KMT2A neg NPM1 neg PML-RARA neg BCR-ABL1 neg CBFB partial del
1st INDUCTION — MEC (Mitoxantrone + Etoposide + Cytarabine) + Intrathecal prophylaxis							
MEC	D + 22	WBC: 480/μL Hb: 7.7 g/dL Plt: 13,700/μL	Sample not viable	4 WBC/μL No blasts	-	4.9%	Myeloid clone present Minor B-clone persists
	D + 29	WBC: 620/μL Hb: 7.25 g/dL Plt: 59,300/μL	1% (CR)	2 WBC/μL No blasts	-	1%	-
	D + 36	WBC: 2,260/μL Hb: 9.3 g/dL Plt: 166,000/μL	0% (CR)	4 WBC/μL No blasts	Decision: proceed to ADE	0.83%	Myeloid clone neg Minor B-clone: CD22++; CD19++
2nd INDUCTION — ADE (Cytarabine + Daunorubicin + Etoposide) + Intrathecal prophylaxis							
ADE	D + 37	-	-	-	NGS sample collected (post-ADE MRD timepoint)	-	TCF3::ZNF384 Fusion read support: 75.04% Tier IA (AMP/ASCO/CAP)
	D + 78	WBC: 6,370/μL Hb: 10.1 g/dL Plt: 158,000/μL	0% (CR)	Neg No blasts	CNS clinically stable	Neg	-
INFECTIOUS COMPLICATIONS							
Aplasia		Nadir CBC	-	-	•RSV respiratory infection •Probable fungal pneumonia •Probable fungal brain infection (compatible MRI images) •Suspected endocarditis •Neutropenic colitis •Gastrointestinal bleeding •Catheter-associated bacteremia due to *P. aeruginosa*	-	-
CONSOLIDATION — NOPHO-AML (Nordic Society of Paediatric Haematology and Oncology AML) 2012 protocol							
HAM (C1)	D + 117	WBC: 3,500/μL Hb: 8.4 g/dL Plt: 169,000/μL	BM 0% (CR)	Neg No blasts	High-dose Cytarabine + Mitoxantrone	Neg	CR
HA3E (C2)	D + 144	WBC: 2,830/μL Hb: 8.3 g/dL Plt: 188,000/μL	BM1% (CR)	Neg No blasts	High-dose Cytarabine + Etoposide	Neg	CR
FLA (C3)	D + 176	WBC: 700/μL Hb: 7.2 g/dL Plt: 51,600/μL	BM 1% (CR)	Neg No blasts	Fludarabine + Cytarabine	Neg	CR
FOLLOW-UP							
Month 8 post-Tx	Current day	WBC: 5,200/μL Hb: 13.2 g/dL Plt: 269,000/μL	CR	Neg No blasts	End of treatment Ongoing surveillance	Neg	CR

BM, bone marrow; CBC, complete blood count; CNS, central nervous system; CR, complete remission; CSF, cerebrospinal fluid; FC, flow cytometry; IT, intrathecal; MRD, minimal residual disease; NGS, next-generation sequencing; PB, peripheral blood; P/LP, pathogenic/likely pathogenic; Tx, treatment; WBC, white blood cell count.

## 3 Materials and methods

### 3.1 Molecular characterization

Genomic DNA and total RNA were extracted from bone marrow aspirate collected after the second induction cycle (ADE), at which point MRD had become undetectable. Next-generation sequencing was performed using the SOPHiA DDM™ Myeloid Plus Solution panel (SOPHiA GENETICS, Switzerland) on an Illumina MiSeq platform. The panel was run in DNA + RNA mode (Interpretation Scope: MYS_plus_v1) for simultaneous detection of single nucleotide variants (SNVs), insertions/deletions (indels), and gene fusions in genes frequently mutated in myeloid malignancies.

Library preparation for DNA employed enzymatic fragmentation followed by hybrid capture enrichment. RNA libraries were prepared using targeted capture for fusion detection. Variant and fusion calling were performed using SOPHiA DDM™ software (v.10.6.0). For gene fusions, the software reports results as fusion read support (% SR, representing the percentage of reads supporting the fusion event out of total reads at the locus), which is the appropriate metric for fusion quantification; variant allele frequency (VAF%) is reserved for SNVs and indels. Unique molecular coverage (UMC) was additionally reported, reflecting the number and percentage of deduplicated unique molecular identifiers (UMIs) supporting the fusion call. A global low-coverage flag was generated by the platform for this sample; however, the TCF3::ZNF384 fusion itself was supported by robust read-level evidence (21,262 total reads; 3,125 UMIs) as detailed in Section 4 and Table 2.

**Table 2:** Molecular findings by next-generation sequencing.

Finding	Result
Gene Fusion	
TCF3::ZNF384 e11::e3 (in-frame)	Detected
Fusion Read Support (% SR)	75.04% (21,262 reads)
Unique Molecular Coverage (UMC)	80.56% (3,125 UMIs)
Classification	Tier IA (AMP/ASCO/CAP)
Supporting Databases	COSMIC, ChimerPub, ChimerSeq
Unique ID	TCF3(NM 003200:11)-ZNF384(NM 001135724:3)
Recurrent AML/ALL Alterations	
FLT3-ITD	Negative
NPM1 mutation	Negative
KMT2A rearrangement	Negative
BCR-ABL1	Negative
RUNX1-RUNX1T1	Negative
CBFB-MYH11	Negative
PML-RARA	Negative
Additional Findings	
Pathogenic/Likely Pathogenic	None
Variants of Uncertain Significance	22*

* A total of 22 VUS (Variants of Uncertain Significance, classified per AMP/ASCO/CAP somatic variant guidelines) were identified across 15 genes: ABL1, ASXL1, CSF3R, EZH2, ETV6, FLT3, JAK2, PTPN11, SETBP1, SF3B1, SRSF2, TET2, TP53, WT1, ZRSR2. None met criteria for pathogenic or likely pathogenic classification in the context of this disease. fusion events are reported as fusion read support (% SR), not as variant allele frequency (VAF%), which is reserved for SNVs and indels.

Variants were classified according to AMP/ASCO/CAP guidelines for somatic variants in cancer, where Tier IA denotes the highest level of clinical actionability based on FDA-approved therapies or professional guideline recommendations in the specific tumor type. FLT3-ITD was assessed by fragment analysis.

Orthogonal confirmation of the TCF3::ZNF384 fusion by a separate assay (e.g., RT-PCR or independent targeted RNA sequencing) was not performed. However, the fusion was detected with high confidence by the SOPHiA DDM panel using simultaneous DNA and RNA analysis, with 75.04% read support and 80.56% unique molecular coverage, supported by entries in COSMIC, ChimerPub, and ChimerSeq databases (Unique ID: TCF3[NM_003200:11]-ZNF384[NM_001135724:3]). This limitation is acknowledged.

Conventional cytogenetics (G-banding) and fluorescence *in situ* hybridization (FISH) for recurrent translocations (KMT2A, RUNX1-RUNX1T1, CBFB-MYH11, PML-RARA) were performed according to standard protocols. Fewer than 20 metaphases were analyzable (reported as inconclusive), with G-banding resolution of approximately 400 bands. FISH was performed using Vysis/Abbott probes (KMT2A break-apart, RUNX1-RUNX1T1 dual-fusion, CBFB break-apart, PML-RARA dual-fusion), scoring ≥ 200 interphase nuclei per probe. Laboratory cutoffs were > 5% for gains and > 10% for deletions.

### 3.2 Flow cytometry and MRD assessment

Multiparameter flow cytometry was performed on a BD FACSCanto II instrument (8-color configuration). Antibodies were sourced from BD Biosciences and Beckman Coulter. Data acquisition and analysis were performed using Infinicyt software (Cytognos, Salamanca, Spain). The gating strategy employed a CD45/SSC backbone for blast identification, with isotype controls and fluorescence-minus-one (FMO) controls for threshold determination. MPO positivity was defined as cytoplasmic staining in > 20% of blasts. B-lineage criteria were defined according to WHO 2022/ICC classification, requiring expression of ≥ 2 B-cell markers (CD19, CD22, CD79a, or CD10) at diagnostic intensity.

MRD assessment was performed with a sensitivity of 10⁻⁴ (0.01%), using a comprehensive panel that included both myeloid (CD33, CD13, MPO, CD117, CD34) and lymphoid (CD19, CD22, CD10) markers to capture potential lineage shifts, which are characteristic of ZNF384-rearranged leukemias. Immunophenotypic profiles were compared between diagnostic and post-treatment evaluations at each timepoint to monitor for lineage plasticity.

### 3.3 Ethical considerations

This case report describes routine clinical practice; formal ethics committee approval was therefore not required (Ley Orgánica 3/2018; GDPR). Written informed consent for publication was obtained from the patient’s legal guardians. All data are fully anonymized. The study was conducted in accordance with the principles of the Declaration of Helsinki.

## 4 Molecular results. discovery of the TCF3::ZNF384 fusion

Given the persistent MRD after two induction cycles, an expanded molecular analysis was performed using the DNA/RNA-based NGS panel. This revealed an in-frame fusion between TCF3 (exon 11) and ZNF384 (exon 3) with a fusion read support of 75.04% (21,262 supporting reads; unique molecular coverage: 80.56%, 3,125 UMIs). The fusion was annotated in COSMIC, ChimerPub, and ChimerSeq databases (Unique ID: TCF3[NM_003200:11]-ZNF384[NM_001135724:3]) and classified as Tier IA (clearly actionable) according to AMP/ASCO/CAP guidelines. A global low-coverage flag was generated by the SOPHiA DDM platform for this sample; however, the fusion-specific read support was robust and exceeded quality thresholds for clinical reporting. No additional pathogenic or likely pathogenic variants were identified in the analyzed genes (Table 2).

The TCF3::ZNF384 fusion—resulting from a t(12;19)(p13;p13.3) translocation—creates a chimeric protein that disrupts transcriptional programs of hematopoietic differentiation, leading to lineage ambiguity and mixed immunophenotypic expression. Although rare in AML, it is well recognized in B-ALL and MPAL-B/myeloid, often associated with low CD10, aberrant CD13/CD33 expression, and a tendency toward lineage switch. Published data suggest that these leukemias may benefit from hybrid AML/ALL treatment regimens and, in selected cases, targeted agents under investigation.

## 5 Discussion

This case underscores the complexity of diagnosing and treating acute leukemias presenting with mixed or evolving phenotypes. Although the initial working diagnosis was AML based on predominant myeloid markers, the eventual detection of a TCF3::ZNF384 fusion—commonly linked to B-lineage or mixed leukemias—highlights the need for an integrative diagnostic approach, as also illustrated by Lin et al. and Au et al. (6, 10). Distinctively, our patient presented with CNS-3 involvement at diagnosis, uncommonly reported in this entity, and achieved sustained remission with intensified myeloid-directed therapy alone without requiring protocol switch, consistent with the subset of cases described by Shi et al. in whom early MRD negativity after induction predicted favorable outcome under AML regimens (11). The dynamic acquisition of B-lineage markers (CD22++, CD19++) on the minor blast clone during induction provides direct *in vivo* evidence of lineage plasticity, reinforcing the need for dual-lineage MRD monitoring throughout treatment.

### 5.1 Immunophenotypic overlap and lineage plasticity

Co-expression of myeloid and B-cell markers can lead to classification ambiguity (3, 5). In this case, the presence of a minority population expressing CD19++ at diagnosis and emergent CD22 expression during treatment strongly suggested lineage plasticity—a hallmark of ZNF384-rearranged leukemias (7). The high fusion read support (75.04%) of the fusion indicates it was an early, clonal event driving leukemogenesis, rather than a subclonal aberration.

Detecting two distinct blast populations at diagnosis with changes during therapy illustrates the dynamic nature of ZNF384-rearranged leukemias and their propensity for clonal evolution and potential lineage switching (12). All of this underscores the biological complexity of this entity and the challenges it poses for both diagnosis, treatment and MRD monitoring (11).

### 5.2 Therapeutic implications and clinical decision-making

Some TCF3::ZNF384–positive leukemias may respond best to combined or sequential AML/ALL regimens (6). In this case, the clinical decision to continue with a myeloid-directed protocol (NOPHO-AML-2012) was based on several factors:

1. The patient never presented with >5% blasts meeting lineage B criteria according to cytological/morphological evaluation during induction.
2. The predominant immunophenotype at diagnosis was myeloid (84% of blasts).
3. Intensified consolidation with high-dose cytarabine-based protocols (HAM, HA3E, FLA) successfully achieved sustained remission with negative MRD.

The favorable response to intensified myeloid-directed consolidation therapy suggests that, in selected cases with TCF3::ZNF384 fusion presenting predominantly with myeloid features, continuation of AML protocols may be appropriate when rigorous MRD monitoring is in place (13).

However, the persistent MRD after initial induction raises the question of whether earlier implementation of lymphoid-directed therapy or hybrid protocols might have expedited response. The emergence of B-lineage markers during treatment highlights the need for adaptive therapeutic strategies that account for the biological plasticity of these leukemias. The decision not to pursue a hybrid AML/ALL protocol in this case was based on several explicit clinical grounds: (i) the TCF3::ZNF384 fusion was only identified after post-ADE MRD evaluation, at which point a second induction had already been completed; (ii) at no point during induction did morphological assessment show >5% blasts unequivocally meeting B-lineage criteria according to the NOPHO-AML-2012 protocol thresholds; and (iii) following MRD negativity after ADE and subsequent intensified consolidation, the clinical trajectory was favorable. Nevertheless, we acknowledge that earlier molecular characterization and prospective consideration of hybrid or ALL-directed regimens in cases with post-induction MRD positivity and emergent B-lineage markers may represent an area for protocol optimization in future patients with TCF3::ZNF384-positive disease.

### 5.3 Novel therapeutic targets

Novel targeted agents represent an emerging therapeutic avenue (9). Recent studies have demonstrated that ZNF384 fusions can activate FLT3 signaling pathways, suggesting potential sensitivity to FLT3 inhibitors such as gilteritinib (9). Although no FLT3-ITD was detected in our patient and only a synonymous FLT3 variant was identified, functional assessment of FLT3 pathway activation was not performed. Future investigation of FLT3 inhibitor sensitivity in ZNF384-rearranged cases may be warranted.

Additionally, studies have shown that ZNF384 fusion proteins may be susceptible to epigenetic modifiers and inhibitors targeting chromatin remodeling complexes, representing potential avenues for future therapeutic intervention (14).

### 5.4 Role of comprehensive molecular testing

This case exemplifies the critical importance of comprehensive NGS panels in pediatric acute leukemia, particularly when conventional testing yields negative or ambiguous results (2, 8). The identification of TCF3::ZNF384 not only reclassified the biological subtype but also informed therapeutic strategy and MRD surveillance approach.

Close MRD monitoring—both by highly sensitive flow cytometry and by molecular techniques—remains pivotal (1, 2). Early detection of blast re-emergence or lineage switch could inform timely therapeutic modifications and potentially improve outcomes in this challenging leukemia subtype. The use of immunophenotypic markers that capture both myeloid and lymphoid features is essential for accurate MRD assessment in ZNF384-rearranged cases (15). Based on the present case and available literature, we propose three practical take-home messages: (1) RNA-based fusion panel testing should be triggered early in any atypical AML presenting with a minor B-lineage blast population, low CD10, and/or CNS disease, given that ZNF384-rearranged leukemias are frequently misclassified; (2) MRD monitoring in cases with lineage plasticity should incorporate both myeloid and lymphoid immunophenotypic markers (including CD19, CD22, and MPO) throughout the entire treatment course, as clonal evolution may render single-lineage MRD strategies inadequate; and (3) the decision to implement a hybrid AML/ALL treatment approach should be considered early when post-induction MRD remains positive and B-lineage marker emergence is documented, rather than deferred to later consolidation phases.

## 6 Conclusion

We describe a pediatric leukemia initially classified as AML with CNS involvement, later found to harbor a TCF3::ZNF384 fusion typically associated with B-ALL and MPAL. This case underscores the importance of comprehensive immunophenotypic and molecular assessment in atypical acute leukemias. Recognition of hybrid genetic profiles like TCF3::ZNF384 can guide tailored therapeutic approaches, ensure rigorous MRD monitoring, and inform timely intervention strategies to mitigate relapse and lineage conversion risks.

The high fusion read support (75.04%) and absence of additional pathogenic variants suggest this fusion was the primary driver of leukemogenesis, emphasizing its potential as both a diagnostic marker and therapeutic target in this challenging disease entity. The successful achievement of sustained remission with intensified myeloid-directed consolidation, coupled with meticulous MRD surveillance, demonstrates that individualized therapeutic strategies based on predominant immunophenotype and molecular characteristics can yield favorable outcomes in selected ZNF384-rearranged cases.

Future studies should focus on defining optimal treatment algorithms for TCF3::ZNF384-positive leukemias, including the potential role of hybrid protocols, targeted agents, and the utility of molecular MRD monitoring in guiding therapeutic decisions.

## Data Availability

The original contributions presented in the study are not publicly available due to ethical and privacy restrictions related to pediatric human genomic data. De-identified data supporting the findings of this study may be made available from the corresponding author upon reasonable request and subject to institutional and ethical approval.

## References

[1] Tomizawa D Tsujimoto SI. Risk-Stratified therapy for pediatric acute myeloid leukemia. Cancers (Basel). (2023) 15(16):4171. 10.3390/cancers15164171 37627199 PMC10452723

[2] ZwaanCM TasianSK AplencR BrodersenLE BuldiniB De MoerlooseB. Diagnosis and management of AML in pediatric patients: consensus recommendations from an international expert panel. Blood. (2025) 147:1532–61. 10.1182/blood.202402790441105890

[3] Hirabayashi S Ohki K Nakabayashi K Ichikawa H Momozawa Y Okamura K. ZNF384-related Fusion genes define a subgroup of childhood B-cell precursor acute lymphoblastic leukemia with a characteristic immunotype. Haematologica. (2017) 102(1):118–29. 10.3324/haematol.2016.151035 27634205 PMC5210242

[4] Li Y Tan Y Yin H Jiang Y Chen L Meng G. Research progress in the pathogenesis and prognosis of ZNF384 fusion subtype acute leukemia. J Shanghai Jiao Tong Univ (Med Sci). (2023) 43(5):631–40. 10.3969/j.issn.1674-8115.2023.05.015

[5] Srinivasan T Sharma P Sachdeva MUS Peyam S KS L Khadwal A. Clinicopathological and immunophenotypic characteristics of series of ZNF384 Re-arranged acute leukemias. Indian J Hematol Blood Transfus. (2024) 40(4):704–9. 10.1007/s12288-024-01755-5 39469177 PMC11512947

[6] Lin N Yan X Cai D Wang L. Leukemia with TCF3-ZNF384 rearrangement as a distinct subtype of disease with distinct treatments: perspectives from A. Case Report and Literature Review. Front Oncol. (2021) 11:709036. 10.3969/j.issn.1674-8115.2023.05.015 34395283 PMC8357369

[7] Dickerson KM Qu C Gao Q Iacobucci I Gu Z Yoshihara H. ZNF384 Fusion oncoproteins drive lineage aberrancy in acute leukemia. Blood Cancer Discov. (2022) 3(3):240–63. 10.1158/2643-3230.BCD-21-0163 35247902 PMC9414741

[8] Lejman M Chalupnik A Chilimoniuk Z Dobosz M. Genetic biomarkers and their clinical implications in B-cell acute lymphoblastic leukemia in children. Int J Mol Sci. (2022) 23(5):2755. 10.3390/ijms23052755 35269896 PMC8911213

[9] Zhao X Wang P Diedrich JD Smart B Reyes N Yoshimura S. Epigenetic activation of the FLT3 gene by ZNF384 fusion confers a therapeutic susceptibility in acute lymphoblastic leukemia. Nat Commun. (2022) 13(1):5401. 10.1038/s41467-022-33143-w 36104354 PMC9474531

[10] Au WY Chow C To KF Ma ESK Yeung ECL Yip W-L. Mixed-phenotype leukemia with TCF3::ZNF384 fusion presenting as an isolated mediastinal mass. Ann Hematol. (2025) 104:2023–5. 10.1007/s00277-024-06042-x 39531055 PMC12031752

[11] Sudutan T Erbilgin Y Hatirnaz Ng O Karaman S Karakas Z Kucukcankurt F. Zinc finger protein 384 (ZNF384) impact on childhood mixed phenotype acute leukemia and B-cell precursor acute lymphoblastic leukemia. Leuk Lymphoma. (2022) 63(12):2931–9. 10.1080/10428194.2022.2095630 35921545

[12] Alexander TB Gu Z Iacobucci I Dickerson K Choi JK Xu B. The genetic basis and cell of origin of mixed phenotype acute leukaemia. Nature. (2018) 562(7727):373–9. 10.1038/s41586-018-0436-0 30209392 PMC6195459

[13] Rubnitz JE Inaba H Dahl G Ribeiro RC Bowman WP Taub J. Minimal residual disease-directed therapy for childhood acute myeloid leukaemia: results of the AML02 multicentre trial. Lancet Oncol. (2010) 11(6):543–52. 10.1016/S1470-2045(10)70090-5 20451454 PMC3171799

[14] Takahashi K Wang F Morita K Yan Y Hu P Zhao P. Integrative genomic analysis of adult mixed phenotype acute leukemia delineates lineage associated molecular subtypes. Nat Commun. (2018) 9(1):2670. 10.1038/s41467-018-04924-z 29991687 PMC6039465

[15] Chiaretti S Messina M Foà R. BCR/ABL1-like acute lymphoblastic leukemia: how to diagnose and treat? Cancer. (2019) 125(2):194–204. 10.1002/cncr.31848 30561755

